# Effect of Additional Amino Acid Replacements on the Properties of Multi-point Mutant Bacterial Formate Dehyderogenase PseFDH SM4S

**DOI:** 10.32607/actanaturae.11665

**Published:** 2022

**Authors:** A. A. Pometun, P. D. Parshin, N. P. Galanicheva, L. A. Shaposhnikov, D. L. Atroshenko, E. V. Pometun, V. V. Burmakin, S. Yu. Kleymenov, S. S. Savin, V. I. Tishkov

**Affiliations:** Bach Institute of Biochemistry, Federal Research Center of Biotechnology of the Russian Academy of Sciences, Moscow, 119071 Russia; Lomonosov Moscow State University, Department of Chemistry, Moscow, 119991 Russia; Innovations and High Technologies MSU Ltd., Moscow, 109559 Russia; Sechenov First Moscow State Medical University, Moscow, 119991 Russia; Koltzov Institute of Developmental Biology of the Russian Academy of Sciences, Moscow, 119334 Russia

**Keywords:** formate dehydrogenase, Pseudomonas sp. 101, catalytic properties, thermal stability, site-directed mutagenesis

## Abstract

Formate dehydrogenase from Pseudomonas sp. 101 bacterium (PseFDH, EC 1.2.1.2) is a research model for the elucidation of the catalytic mechanism of 2-oxyacid D-specific dehydrogenases enzyme superfamily. The enzyme is actively used for regeneration of the reduced form of NAD(P)H in chiral synthesis with oxidoreductases. A multi-point mutant PseFDH SM4S with an improved thermal and chemical stability has been prepared earlier in this laboratory. To further improve the properties of the mutant, additional single-point replacements have been introduced to generate five new PseFDH mutants. All new enzymes have been highly purified, and their kinetic properties and thermal stability studied using analysis of thermal inactivation kinetics and differential scanning calorimetry. The E170D amino acid change in PseFDH SM4S shows an increase in thermal stability 1.76- and 10-fold compared to the starting mutant and the wild-type enzyme, respectively.

## INTRODUCTION

NAD^+^-dependent formate dehydrogenase (FDH, EC 1.2.1.2) from methylotrophic bacterium Pseudomonas sp. 101 (PseFDH) is one of the best studied enzyme in the group. PseFDH is the first formate dehydrogenases obtained in a highly purified form and characterized [1]. The gene coding for the enzyme, psefdh, has been the first bacterial formate dehydrogenase gene cloned and overexpressed in Escherichia coli [2, 3]. Crystal structures for apo- and holo-forms of PseFDH have been determined (PDB2NAC, PDB2NAD, PDB2GO1, and PDB2GUG structures). Despite the fact that many novel formate dehydrogenases have been cloned, isolated, and characterized in the last decades, PseFDH is still the one with the highest thermal stability [4], and high catalytic activity and efficiency [5, 6]. Formate dehydrogenase from pathogenic bacterium Staphylococcus aureus (SauFDH) has been recently isolated and crystallized in this laboratory [7]; this enzyme is comparable to PseFDH in its thermal stability [4] and exhibits a higher catalytic activity, but not efficiency [6].

We systematically study structure-function relationships in formate dehydrogenases. The importance of His332-Gln313 pair and Arg284 residue in the catalytic mechanism of PseFDH has been confirmed [8, 9]. Hydrophobization of alpha-helices with single-point replacement resulted in the production of mutant forms with improved thermal stability [10]. The experiments aimed at changing the coenzyme specificity have been initiated [11]; the mutants with changed isoelectric point have been constructed [12]. The effect of N-terminal His-tag on the properties of the wild-type enzyme and its NADP^+^-specific mutants has been studied by site-directed mutagenesis [13]. Chemical stability of PseFDH has been improved as well, and the mutants with an increased stability in the presence of hydrogen peroxide have been produced [14, 15].

As seen from above, to construct a novel biocatalyst with just one improved parameter, one needs to introduce a set of amino acid replacements. In some cases, combination of mutations results in synergy. For example, such effect has been observed while improving thermal stability of soybean FDH [16]. In case of PseFDH SM4S mutant, replacements in 311th position generate enzymes with a 2.4-fold improved thermal stability with respect to the initial mutant, and more than 7-fold if compared to the wild-type PseFDH [17].

By combining replacements improving catalytic activity, as well as thermal and operational stability, we have generated PseFDH SM4S variant. Here we continued experiments to improve properties of the above mutant. Additional single-point amino acid substitutions were introduced in PseFDH SM4S. Previously it have been shown that these changes provided positive effect on the properties of the wild-type enzyme.

## EXPERIMENTAL

**Site-directed mutagenesis**

Single point amino acid substitutions were introduced using a two-step polymerase chain reaction (PCR). The pPseFDH8_SM4S plasmid, with psefdh gene under control of a strong T7 phage RNA polymerase promoter, was used as a template. To introduce mutations, forward (T7_for) and reverse (T7_rev) primers for the gene ends, as well as forward and reverse primers carrying the required substitution in the psefdhsm4 gene were used:

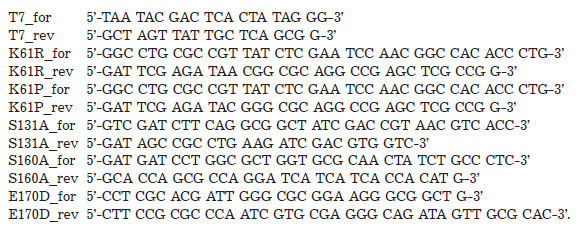



The PCR reaction mixture contained 2.5 μL of 10x Pfu DNA polymerase buffer (200 mM Tris-HCl (pH 8.8 at 25°C), 100 mM (NH_4_)_2_SO_4_, 100 mM KCl, 1 mg/mL BSA, 1% (v/v) Triton X-100, 20 mM MgSO_4_); 2.5 μL of dNTP mix (dATP, dGTP, dTTP, dCTP, concentration of each 2.5 mM); 1 µL DNA template (≈10 ng/µL); 2 µL of primers (10 nmol/mL); 0.5 µL of Pfu DNA polymerase (2.5 U/µL) and deionized water to a total mixture volume of 25 µL. PCR was performed in a 0.5 mL thin-walled plastic tube (SSI, USA) on a Tertsik device ("DNA-Technologies", Russia).

prevent evaporation of the reaction mixture, 30 µL of mineral oil was added to the tube. The tube was heated for 5 min at 95°C and then the reaction was carried out according to the following program: denaturation, 95°C, 30 s; primer binding, 54–58°C; elongation, 72°C, 2 min, 25–35 cycles in total. After the last cycle, the reaction mixture was additionally left for 5 min at 72°C. The temperature at the second stage was chosen to be 3–5°C lower than the melting temperature of duplexes (Tm) formed by the primers.

To obtain fragments containing the desired substitution, two PCRs were performed using primer pairs: 1) forward PseFor containing the required nucleotide(s) substitution(s) and reverse standard primer T7_rev (fragment 1); standard forward primer T7_for and reverse primer PseRev, also containing the required nucleotide(s) substitution(s) (fragment 2). The products of two PCRs were purified by electrophoresis in 1% agarose gel followed by isolation of DNA fragments from the gel. At the next stage, the third combining PCR was performed with primers T7_for and T7_rev, where both previously obtained fragments were used as a DNA template.

The product of the third PCR was purified in the same manner and then digested with restriction endonucleases NdeI and XhoI. The PseFDH_SM4S plasmid was treated with the same restrictases to remove the gene fragment with the introduced mutation. The digested PCR product and plasmid were purified by electrophoresis and ligated. The mixture obtained after the ligation reaction was transformed into E. coli DH5α cells. The introduction of the required mutations was controlled by plasmid DNA sequencing at the Genome Center of Collective Use, Engelhardt Institute of Molecular Biology, Russian Academy of Sciences or at the Industrial Biotechnology Center of Collective Use, Federal Research Center for Biotechnology, Russian Academy of Sciences.

**Expression of new PseFDH mutants in E. coli cells**

PseFDH wild-type and mutant variants were expressed in E. coli BL21(DE3)/pLysS cells. Cells were transformed with the corresponding plasmid and plated on Petri dishes with agar medium containing ampicillin (100 μg/mL) and chloramphenicol (25 μg/mL). To prepare the inoculum, a single colony was taken from the dish and cultured in 5 mL of 2YT medium (yeast extract 10 g/L, bactotrypton 16 g/L, sodium chloride 5 g/L, pH 7.0) in the presence of 150 μg/mL ampicillin and 25 μg/mL chloramphenicol for 7–9 hrs at 30°C and 180 rpm until the absorption at a wavelength of 600 nm was A600 ≈ 0.6–0.8. Then, 2 mL of the overnight culture were transferred into 100 mL shaked flasks containing 20 mL of 2YT medium and 150 µg/mL ampicillin and the cells were cultured at 37°C and 120 rpm until the absorbance of A600 = 0.6–0.8 was reached. Then cells were reseeded into flasks containing 230 ml of 2YT medium without antibiotics and cultivated at 30°C until the absorbance value A600 ≈ 0.6–0.8. Protein synthesis was induced by adding a lactose solution (300 g/L) to the culture medium to a final concentration of 20 g/L. After induction, the cells were incubated for 17 hrs at 120 rpm and 30°C. The cell biomass after cultivation was collected on a Beckman J-21 centrifuge (USA) at 7500 rpm for 20 min at 4°C. The supernatant was removed and the cells were resuspended in 0.1 M sodium phosphate buffer pH 8.0 in a ratio of 1 : 4 (w/w). The resulting suspension was frozen and stored at –20°C.

**Isolation and purification**

Cells after cultivation were disintegrated by sonication. Cellular debris was precipitated by centrifugation (Eppendorf 5804R, 40 min, +4°C, 12000 rpm), and a saturated solution of ammonium sulfate was added to the supernatant to final concentration 35% of saturation (0.1 M sodium phosphate buffer, 0.01 M EDTA, pH 7.0 (solution A)) and the final solution was incubated for 4–8 h at +4°C. Undissolved proteins were precipitated in 50 ml tubes on an Eppendorf 5804 R centrifuge (11,000 rpm, +4°C), and the resulting supernatant was applied to a 1.0 × 10 cm column with Phenyl Sepharose FastFlow (Pharmacia Biotech, Austria) equilibrated with solution A. After applying the enzyme, the column was washed with solution A until absorption at 280 nm disappeared. The enzyme was eluted from the column with a linear ammonium sulfate gradient (35–0% saturation, 0.1 M phosphate buffer, 0.01 M EDTA, pH 7.0, total volume 150 mL). 5 mL fractions were collected, absorbance at 280 and 260 nm (A_280_ and A_260_, respectively) and enzymatic activity (A) were measured. Fractions with the maximum ratio (A/A_280_) were combined. Desalting was performed on a 2.5 × 10 cm column (volume 25 mL) with Sephadex G25 (Pharmacia Fine Chemicals, Sweden) equilibrated with 0.1 M Na-phosphate, 0.01 M EDTA, pH 7.0. Fractions of 0.5 mL were collected, and enzymatic activity and absorbance at 280 nm were determined in each fraction. The purity of the preparations was controlled by analytical electrophoresis in 12% polyacrylamide gel in the presence of 0.1% sodium dodecyl sulfate on a BioRad MiniProtean II electrophoresis device according to the manufacturer’s protocol. The enzyme concentration in the samples was calculated from the absorption value of 1.6 for a 0.1% solution of purified PseFDH at a wavelength of 280 nm.

**Measurement of formate dehydrogenase activity**

FDH activity was determined spectrophotometrically by the accumulation of NADH (NADPH) at a wavelength of 340 nm (ε_340_ = 6,220M^–1^ cm^–1^) on a Schimadzu UV1800 PC spectrophotometer at 30°C in 0.1 M sodium phosphate buffer, pH 7.0. The concentration of sodium formate and NAD(P)^+^ in the cuvette was 0.6 M and 1 mg/mL, respectively.

**Determination of Michaelis constants**

The Michaelis constants for NAD^+^ and formate were determined from the dependences of the enzyme activity on the concentration (0.4–6 K_M_) of the corresponding substrate. The concentration of the second substrate was saturating (>15 K_M_). The exact concentration of the stock NAD^+^ solution was determined spectrophotometrically at a wavelength of 260 nm (ε_260_ = 17,800 M^-1^cm^-1^).

A solution of sodium formate with a given concentration was prepared by dissolving the required amount of the substrate in 0.1 M sodium phosphate buffer, pH 7.0. The volume of the solution was controlled in a volumetric flask. The KM values were calculated from the experimental dependences by the method of non-linear regression using the Origin Pro 2015 program.

**Thermal inactivation kinetics**

Thermal stability of enzymes was measured in 0.1 M sodium phosphate buffer, pH 7.0 at several temperatures. Test tubes (0.5 mL volume) with 100 μL of the enzyme solution (0.2 mg/mL) were placed in a water thermostat preheated to the required temperature (temperature control accuracy ±0.1°C). At certain time points, one tube was taken and transferred to ice for 5 min, then the tube was centrifuged for 3 min at 12,000 rpm in an Eppendorf 5415D centrifuge. A residual FDH activity was measured in triplicate as described above. The thermal inactivation rate constant (k_in_) was determined as the slope of the direct dependence of the natural logarithm of the residual activity on time (semilogarithmic coordinates ln(A/A_0_) – t) by linear regression using the Origin Pro 8.1 program.

**Determination of temperature stability by differential scanning calorimetry**

Temperature stability was studied on a Nano DSC differential adiabatic scanning microcalorimeter (TA Instruments, USA). The working volume of capillary calorimetric platinum cells was 300 µL. To prevent the formation of bubbles and boiling of solutions with increasing the temperature, an excess pressure of 3 atm was maintained in the cells of the calorimeter. Before the experiment, the instrumental baseline was recorded and then subtracted from the data obtained for the protein. During measurements, a buffer solution was placed in the control cell, and FDH solution in the same buffer was placed in the working cell. The enzyme concentration was 1–2 mg/mL, and the heating rate was 1°C/min.

## RESULTS AND DISCUSSION

**Selection of residues for directed mutagenesis**

C145S, C255A, and A198G mutations are the key amino acid replacements in PseFDH SM4S mutant. The first two protect the active site of PseFDH from chemical modification and/or oxidation of essential cysteine residues. The C255A replacement results in preservation of 60% of the enzyme activity after 90-day storage at 25°C, whereas the wild-type PseFDH becomes completely inactive at this point [14]. The double replacement C145S/C255A decreases the enzyme inactivation rate constant in the presence of 100 mM hydrogen peroxide by almost 100 times [15]. The A198G replacement provides a decrease in structural tension in the polypeptide chain turn connecting βA beta-sheet and αB helix in the coenzyme binding domain of PseFDH active site. This replacement improves the enzyme thermal stability 2.6-fold, and Michaelis constant for NAD^+^ almost 2 times [11]. The present work focuses on the improvement of the thermal stability of PseFDH SM4S mutant.

**Lys61 replacements**

A shift in the medium pH from 7.0 to 8.0 increases the rate constant of PseFDH thermal inactivation by 6 times [18]. This fact may be interpreted by ionic pairs disruption in alkaline pH, for example, by losing the positive charge on ε-amino group of lysine residue. In the previous work on FDH from Mycobacterium vaccae N10 (MycFDH), which has 4 times worse thermal stability than PseFDH but differs from PseFDH by two amino acid residues, with one being Glu61 instead of K61 in PseFDH, the introduced mitations Glu61K (like in PseFDH) or Glu61Pro yield mutant MycFDHs close in their stability to PseFDH [19]. The analysis of apo- and holo-PseFDH structures (PDB2NAC and PDB2NAD, respectively) points to the ionic pair formed by K61 amino group and Asp43 carboxyl. The same stabilization effect of the introduced Pro61 to that of Lys61 means that the ionic pair, responsible for the support of the enzyme structural stability, is preserved in the K61P mutant with the increase in pH. Additionally, K61R replacement can be introduced, since guanidine group will preserve the positive charge up to at least pH 12.

**Hydrophobization of S131 and S160 residues**

An approach to proteins stabilization based on hydrophobization of protein α-helices, is known for a while [20]. The most frequent Ser/Ala replacement in α-helices is a universal and effective approach for majority of proteins. For example, using this approach, we increased the thermal stability of D-amino acid oxidase by several fold [21]. The analysis of PseFDH structure revealed five Ser residues located in α-helices, among which only one was conserved. Mutations of the other four residues showed that the highest stabilization effect (ca. 20% each) was observed for S131A and S160A replacements [10]. These particular replacements were selected for introduction into PseFDH SM4S.

**Glu170Asp replacement**

**Fig. 1:**
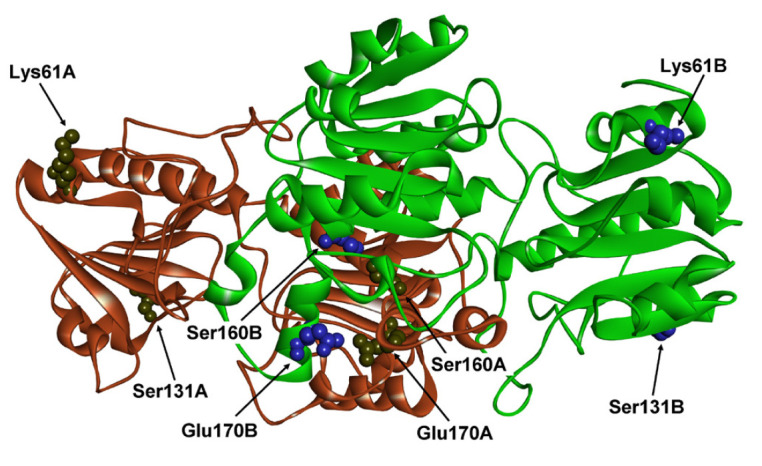
Positions of Lys61, Ser131, Ser160 and Glu170 in the structure of apo-form of FDH from *Pseudomonas *sp. 101 (PDB2NAC)

The Glu170 residue is located at the center of the protein globule, at the subunit interface (Fig. 1), and the negatively charged oxygen atoms of Glu170A carboxyl group of the first subunit are only 2.67 Å far from the oxygen atoms of Glu170B carboxyl group of the second subunit. A removal of carboxylic groups is inappropriate, since these groups participate in electrostatic interactions, and in particular with Arg173 guanidine groups from both A and B subunits (2.64 Å distance). To decrease the mutual repulsion of Glu170 residues without disrupting the whole system of Glu170 interactions, Asp residue can be introduced, because it is shorter by one CH_2_-gropup than Glu residue [22]. Of note, Moraxella sp. C2 FDH (84% homologous to PseFDH) does have Asp residues in position 170 [23, 24]. The E170D replacement in PseFDH resulted in a 40% increase in the enzyme thermal stability [22].

Thus, the structural analysis allowed us to choose 5 amino acid replacements in 4 positions for directed mutagenesis as shown in Fig. 1. Of note, they are located both on the surface and inside the protein globule, including the subunit interface, which is not accessible for solvent molecules. Each replacement in the wild-type PseFDH did not give a significant improvement in stability (max to 40%), however, our previous mutagenesis experience let us expect synergy for the introduced replacements, and the combined effect could be sufficiently high.

**Production of PseFDH SM4S mutant forms**

**Fig. 2:**
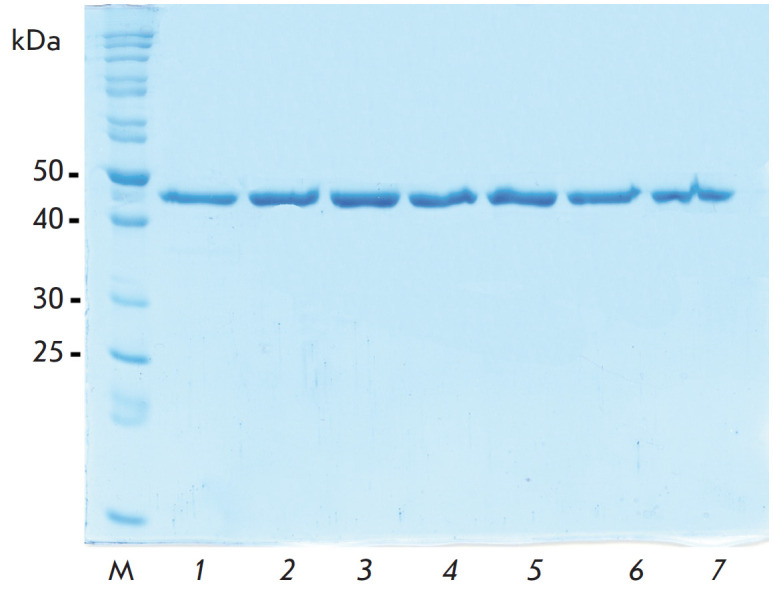
Analytical electrophoresis in 12% polyacrylamide gel in the presence of SDS-Na of enzyme preparations after purification. M – molecular weight marker; 1 – PseFDH wild type; 2 – PseFDH SM4S; 3 – PseFDH SM4S K61P; 4 – PseFDH SM4S E170D; 5 – PseFDH SM4S K61R; 6 – PseFDH SM4S S131A; 7 – PseFDH SM4S S160A

New mutants of PseFDH SM4S have been constructed in accordance with the protocol described in the Experimental Section. Gene sequencing shows that only target mutations have been introduced. The expression results are summarized in Table 1. Expression of PseFDH SM4S and wild-type enzyme PseFDH are used as control. The data obtained allow us to conclude that the protocol developed for wild-type PseFDH expression is applicable for production of all new enzyme forms. Isolation and purification of mutant PseFDH forms has been performed using the method developed by us earlier [8]. The results of analytical electrophoresis of PseFDH mutants shown in Fig. 2 confirm that each preparation contains only one lane and no impurities. Thus, the enzyme preparations obtained are no less than 99% pure.

**Table 1:** Expression of mutant forms of PseFDH and wild-type enzyme in E. coli cells

Enzyme	Enzyme yield, activity, U/L of medium	Biomass yield, g/L of medium	Yield of enzyme by mass^*^, mg/L of medium	Enzyme content in cells, U/g
PseFDH wt	3875	13.5	388	287
PseFDH SM4S	5430	12.0	543	462
PseFDH SM4S K61P	4865	17.0	487	300
PseFDH SM4S K61R	4575	17.2	458	265
PseFDH SM4S S131A	5200	20.0	520	213
PseFDH SM4S S160A	5450	17.0	545	315
PseFDH SM4S E170D	6300	17.0	630	358

^*^Enzyme yield per 1 liter of medium was calculated based on activity yield (column 2) and specific activity value of 10 U/mg of protein.

**Table 2:** Kinetic parameters of mutant PseFDHs and wild-type enzyme

Enzyme	k_cat_, s^-1^	K_M_^HCOO-^, mM	K_M_^NAD+^, µM	k_cat_/K_M_^NAD+^, (M^-1^s^-1^)×10^6^	k_cat_/K_M_^HCOO-^, (M^-1^s^-1^)×10^3^
PseFDH wt	7.3 ± 0.3	1.63 ± 0.08	52.5 ± 2.5	0.14	4.47
PseFDH SM4S	7.3 ± 0.3	1.36 ± 0.14	35.5 ± 1.5	0.21	5.37
PseFDH SM4S K61P	7.3 ± 0.3	1.19 ± 0.08	48.3 ± 1.7	0.15	6.13
PseFDH SM4S K61R	7.7 ± 0.4	1.89 ± 0.11	45.8 ± 2.0	0.17	4.07
PseFDH SM4S S131A	7.5 ± 0.4	2.31 ± 0.15	48.6 ± 1.6	0.15	3.25
PseFDH SM4S S160A	7.3 ± 0.3	1.22 ± 0.12	48.6 ± 2.7	0.15	5.98
PseFDH SM4S E170D	7.3 ± 0.3	1.11 ± 0.08	41.0 ± 1.7	0.18	6.58

Note. 0.1 M sodium phosphate buffer, 0.01 M EDTA, pH 7.0, 30°C.

**Kinetic properties of enzyme mutants**

The values of catalytic and Michaelis constants for NAD^+^ and HCOO-for all PseFDH mutants obtained are summarized in Table 2. Of note, the apparent value of the catalytic constant for all PseFDH mutants remains unchanged within the experimental error. A small increase in Michaelis constant for formate is observed for S131A change (60% and 40% as compared to PseFDH SM4S and wild-type PseFDH, respectively). The K61R replacement has a similar effect on K_M_ for formate. The values of Michaelis constants for NAD^+^ for the mutants obtained remain unchanged within the experimental error (a 10–20% increase and 15–35% decrease in comparison with K_M_ for PseFDH SM4S and wild-type PseFDH, respectively). As a consequence of these subtle changes, the catalytic efficiency k_cat_/K_M_^NAD^+^^ for all mutants is 1.4-fold lesser than that for PseFDH SM4S and equals to that of wild-type PseFDH. The value of kcat/K_M_^HCOO-^is slightly increased as the result of K61P and S160A replacements in PseFDH SM4S. Overall, the introduced replacements do not cause noticeable effects on the enzyme catalytic properties.

**Thermal stability of PseFDH mutant forms**

**Fig. 3:**
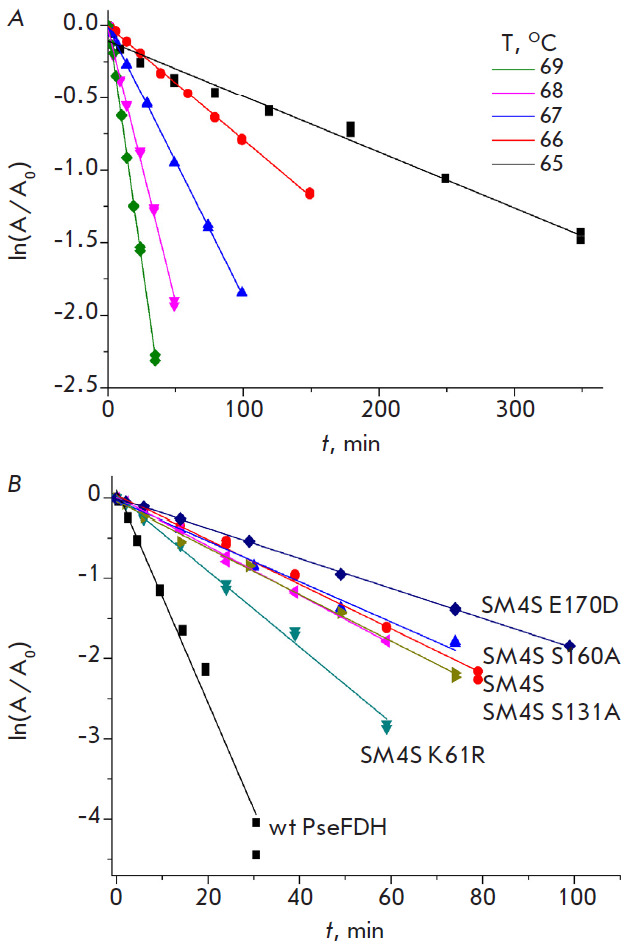
(*A*) Dependence of the residual activity of PseFDH SM4S E170D on time in coordinates ln(A/A_0_) – t, at several temperatures. (*B*) Time dependence of the residual activity of wild-type PseFDH and its mutant forms in coordinates ln(A/A_0_) – t (s) at 67°C, 0.1 M sodium phosphate buffer, pH 7.0

Thermal stability of PseFDH mutants has been studied in the temperature range of 65–69°C, where thermal inactivation of the wild-type enzyme proceeds irreversibly in accordance with a monomolecular mechanism and first-order reaction kinetics [19]. An example of a dependence of the enzyme residual activity on time in a semi-logarithmic coordinates is shown in Fig. 3A for PseFDH SM4S E170D. As seen, the semi-logarithmic plot shows a linear dependence, and thus, the inactivation process obeys first-order re action kinetics. The apparent first-order rate constant for thermal inactivation, kin, is calculated from the slope of the linear dependence. The residual activity at 67°C for all enzyme forms studied plotted versus time in the semi-logarithmic coordinates is shown in Fig. 3B. It is clear that the highest stabilization effect is observed for E170D replacement in PseFDH SM4S. The K61R replacement slightly destabilizes PseFDH SM4S, however, the K61R PseFDH SM4S is still more stable than the wild-type enzyme. All other mutants exhibit the stability similar to that of PseFDH SM4S (Fig. 3B).

The analysis of the temperature dependence of the apparent rate constant of thermal inactivation gives an answer to the thermodynamic origin of the improved stability of PseFDH SM4S E170D mutant. The true monomolecular character of PseFDH inactivation in the whole range of the temperatures studied allows us to apply the transition state theory for the analysis of the inactivation process.

According to the theory, the equation for the apparent rate constant of thermal inactivation has the following dependence on the temperature:

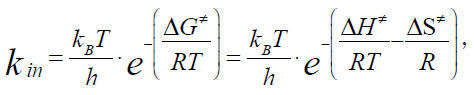



where k_B_ = 1.238 × 10^-23^ J/K – is Bolzmann’s constant; h = 6.634 × 10^–34^ J/s^-1^ – is Plank’s constant; R = 8.314 J/mol/K – is universal gas constant.

The linearized equation looks as:

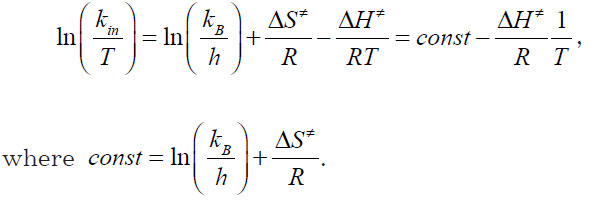



**Fig. 4:**
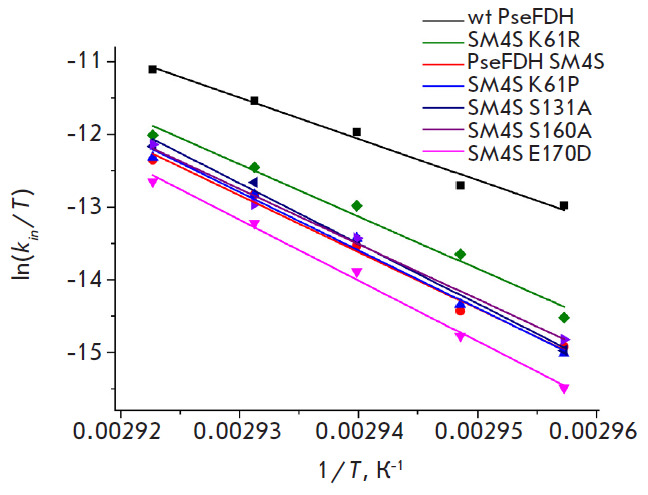
Temperature dependences of the observed thermal inactivation rate constants for wild-type and mutant PseFDHs in coordinates ln(*kin*/T) – 1/T. 0.1 M sodium phosphate buffer, pH 7.0

The dependence in ln(k_in_/T) – 1/T coordinates is linear with the slope equal to -ΔH^≠^/R. The experimental data on the dependence of the apparent rate constant for thermal inactivation for all PseFDH mutants are plotted in Fig. 4. As one can see, in all cases the character of the dependence is the same as for PseFDH SM4S. Using the transition state theory, the values of enthalpy (ΔH^≠^) and entropy (ΔS^≠^) have been calculated. The value of ΔS^≠^ can be obtained from the slope of the dependence of ΔG^≠^ on temperature in accordance with the equation:

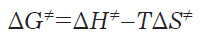



The value of activation Gibbs energy can be calculated from:

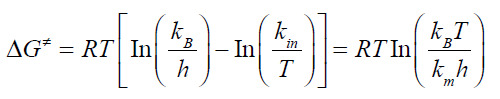



As seen in Table 3, ΔH^≠^ and ΔS^≠^ for the mutants and PseFDH SM4S have the close values and they are higher than for the wild-type enzyme. The highest value of ΔH^≠^ is observed for the mutant with the highest stabilization effect, PseFDH SM4S E170D (Table 3).

**Table 3:** Parameters for thermal inactivation process of mutant PseFDHs and wild-type enzyme

Enzyme	ΔH^≠^, kJ/mol	ΔS^≠^, J/mol/K	ΔH_DSC_, kJ/mol	Phase transition temperature, T_m_, °C
PseFDH wt	470 ± 35	1100 ± 100	1470	68.3
PseFDH SM4S	650 ± 40	1600 ± 100	1975	70.9
PseFDH SM4S K61P	665 ± 40	1650 ± 100	1880	70.9
PseFDH SM4S K61R	600 ± 40	1450 ± 100	nd	nd
PseFDH SM4S S131A	630 ± 50	1720 ± 100	nd	nd
PseFDH SM4S S160A	690 ± 35	1550 ± 100	nd	nd
PseFDH SM4S E170D	700 ± 30	1730 ± 100	2070	71.4

Note. nd – no data. 0.1 M sodium phosphate buffer, pH 7.0.

**Fig. 5:**
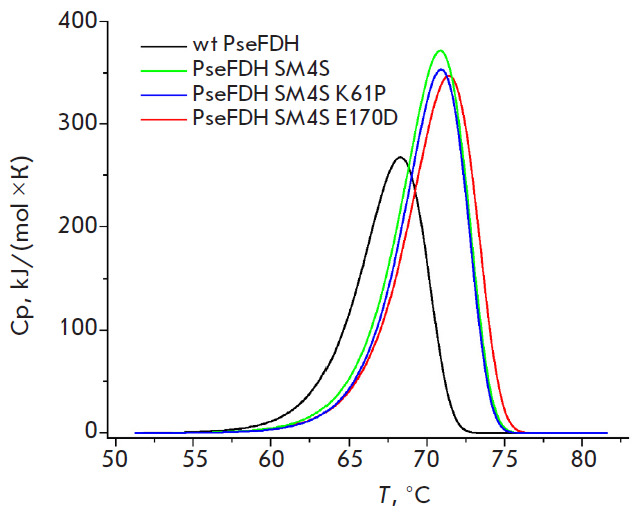
DSC melting curves for mutant and wild-type PseFDH, 0.1 M sodium phosphate buffer, 0.01 M EDTA, pH 7.0. Protein concentration 2 mg/mL, scanning speed 1 °C/min

Thermal stability of some most interesting PseFDH mutant forms has been also studied using differential scanning calorimetry. Since the mutants exhibit similar stability, the above method has been used for the wild-type enzyme, PseFDH SM4S and two its variants with additional replacements E170D and K61P (S131A and S160A exhibit close stabilization effects), as shown in Fig. 5. The numeric values of the heat capacity and phase-transition temperature calculated from the melting curves are shown in Table 3. As seen from Table 3 and Fig. 5, E170D replacement results in the highest increase in the maximum temperature at the melting curve (0.5°C) as compared to the one for PseFDH SM4S. Such increase is in agreement with the magnitude of the stabilization effect observed in kinetic experiments on thermal inactivation. The E170D substitution also causes an increase in the specific heat of phase transition in comparison with the other mutants studied and the wild-type enzyme (Table 3), being in agreement with the analysis of thermal inactivation kinetics with the help of the transition state theory. Thus, there is a good agreement in the results obtained by two independent approaches to the study of thermal stability of PseFDH mutants.

## CONCLUSION

The values of relative thermal stability of the newly obtained mutants with respect to the wild-type PseFDH and the starting PseFDH SM4S mutant (value in brackets) at different temperatures are shown in Table 4. The comparison of the stabilization effects leads us to a number of conclusions.

**Table 4:** Values of the stabilization effect* of mutant enzymes with respect to wild-type PseFDH and PseFDH SM4S at various temperatures

Enzyme	Stabilization effect, k_in_^wt^/k_in_^mut^(k_in_^SM4S^/k_in_^mut^)				
	Temperature, °C				
	65	66	67	68	69
PseFDH wt	1.0	1.0	1.0	1.0	1.0
PseFDH SM4S	7.03(1.0)	5.59(1.0)	4.86(1.0)	3.78(1.0)	3.62(1.0)
PseFDH SM4S K61R	4.43(0.63)	2.58(0.56)	2.75(0.57)	2.5(0.59)	2.48(0.61)
PseFDH SM4S K61P	7.70(1.1)	5.16(0.92)	4.30(0.90)	3.5(0.97)	3.4(0.97)
PseFDH SM4S S131A	7.39(1.05)	4.13(0.93)	4.5(0.93)	4.2(1.15)	2.9(0.83)
PseFDH SM4S S160A	6.35(0.90)	4.13(0.93)	4.3(0.90)	3.1(0.95)	2.81(0.81)
PseFDH SM4S E170D	12.40(1.76)	7.95(1.41)	6.85(1.42)	5.4(1.49)	4.7(1.35)

^*^Stabilization effect calculated as the ratio of the observed inactivation rate constant of the mutant enzyme to the observed inactivation rate constant of wild-type PseFDH at a given temperature (k_in_^wt^/k_in_^mut^). Values in parentheses show the corresponding k_in_^SM4S^/k_in_^mut^ ratios, in which the observed rate constant of thermal inactivation of PseFDH SM4S was taken as the baseline.

0.1 M sodium phosphate buffer, 0.01 M EDTA, pH 7.0.

1. Replacements in position 61 confirmed the importance of K61 residue in supporting the active PseFDH structure. Despite the fact that K61R substitution results in destabilization of PseFDH SM4S, the additional mutation still results in the variant with a higher stability that the wild-type. As mentioned above, the replacement of Lys61 with Pro aimed at the removal of the ionic pair without compromising the stability. The results obtained confirm the validity of our hypothesis at pH 7.0. It has been also proposed that the removal of the ionic pair could increase the enzyme thermal stability at an increased pH of 8.0. Preliminary experiments show that K61P change in PseFDH SM4S at pH 8.0 and the standard 0.1 M phosphate buffer actually results in a decrease in the apparent thermal inactivation rate constant, in comparison with the starting PseFDH SM4S enzyme form. The further work will be performed in a wider range of buffer concentrations, because A198G substitution results in the change of the profile of the dependence of the thermal inactivation rate constant on the buffer concentration compared to the wild-type enzyme [18].

2. Replacements S131A and S160A cause no measurable change in the thermal stability of PseFDH SM4S. This is likely the result of the mild stabilization effect even for the wild-type enzyme (no more than 20%), which is negligibly small and falls within the experimental error when one studies PseFDH SM4S, which thermal stability at 65–69°C is 3.6–7.0 times higher than for the wild-type PseFDH.

3. The E170D replacement in the highly stable PseFDH SM4S results in a 2-fold stronger stabilization effect than that for the wild-type enzyme, hence, we do observe a strong synergy effect (200%). In addition, a higher enthalpy of activation (ΔH≠ = 700 and 470 kJ/mol for PseFDH SM4S E170D and wild-type PseFDH, respectively) leads to the values of apparent rate constants of the new mutant enzyme inactivation at application temperatures (25–40°C) thousand times lesser than those for the wild-type enzyme. The mutant obtained at these temperatures will be hundreds times more stable than the starting PseFDH SM4S mutant.

4. Since four single-point substitutions, e.g. K61P, S131A, S160A, E170D, do no change or even slightly increase thermal stability, and barely affect the kinetic parameters in comparison with PseFDH SM4S, they open a possibility of combining all the amino acid changes into a multi-point mutant. Strong synergy observed upon introducing E170D replacement into PseFDH SM4S supports the need in producing multi-point mutants, because by analogy, one may expect a similar synergic effect for combination of the other three mutations. Preliminary modeling and calculations (to be published in separate) demonstrate that there is one more possible replacement that could improve chemical stability and Michaelis constants for both NAD^+^ and formate. The work in this direction is underway.

## Abbreviations

FDH: formate dehydrogenase
NAD(P)+: nicotinamide adenine dinucleotide (phosphate).
